# Agarose Gel Electrophoresis Reveals the Molecular Weight Distribution of Hyaluronan Produced by Orbital Fibroblasts

**DOI:** 10.3390/gels11060406

**Published:** 2025-05-29

**Authors:** Erika Galgoczi, Monika Katko, Sara Borbely, Istvan Orsos, Zsanett Molnar, Bernadett Ujhelyi, Zita Steiber, Endre V. Nagy

**Affiliations:** 1 Division of Endocrinology, Department of Internal Medicine, Faculty of Medicine, University of Debrecen, Nagyerdei körút 98, 4032 Debrecen, Hungary; galgoczi.erika@med.unideb.hu (E.G.); katko.monika@med.unideb.hu (M.K.); saraborbely@gmail.com (S.B.); orsosistvan99@gmail.com (I.O.); mzsanett82@med.unideb.hu (Z.M.); 2 Doctoral School of Health Sciences, University of Debrecen, 4032 Debrecen, Hungary; 3 Department of Ophthalmology, Faculty of Medicine, University of Debrecen, Nagyerdei körút 98, 4032 Debrecen, Hungary; bujhelyi@med.unideb.hu (B.U.); zsteiber@gmail.com (Z.S.)

**Keywords:** TED, hyaluronan, HMW-HA, MMW-HA, LMW-HA, gel electrophoresis

## Abstract

Thyroid eye disease (TED) is characterized by autoimmune inflammation and structural remodelling of orbital tissues, which is a consequence of the activation of orbital fibroblasts (OFs). As a result of this activation, the production of hyaluronan (HA) and the proliferation and adipocyte differentiation of OFs are enhanced. Adipogenesis leads to additional accumulation of HA. The aim of this study was to elucidate the molecular weight distribution of HA produced by OFs under basic conditions and after adipogenic stimuli. The concentration and the molecular weight distribution of HA were examined using ELISA and agarose gel electrophoresis, respectively, in TED (n = 3) and non-TED (n = 3) OF cultures. Under adipogenic stimuli, HA production is increased in OFs. In TED OF cultures, which, unlike non-TED OFs, can differentiate into adipocytes, the enhanced proportion of high-molecular-weight (HMW) HA of more than 2000 kDa is responsible for the increased HA concentration in the culture media. In non-TED OF cultures, which contain a negligible number of differentiating cells after adipogenic stimulation, the medium-molecular-weight (MMW) HA fragments from 50 to 1000 kDa also contribute to the enhanced HA content. Increased production of HMW-HA during adipocyte differentiation of TED OFs is responsible for the elevated HA content in the culture media, which may be an important contributor to both connective tissue matrix expansion and edema in the pathogenesis of TED.

## 1. Introduction

Thyroid eye disease (TED) is characterized by autoimmune inflammation and orbital connective tissue remodelling [1]. The autoimmune process responsible for the hyperthyroidism and activation of orbital fibroblast (OFs) is the production of autoantibodies against the thyroid stimulating hormone receptor (TSHR) [2], which increases the proliferation, hyaluronan (HA) production, and adipogenic differentiation of OFs [3]. Enhanced HA production is a known accompanying phenomenon of adipocyte differentiation [4]. HA is a major component of the extracellular matrix (ECM), and it has a role in cell–cell and cell–ECM interactions, proliferation, and signal transduction [5]. HA is a polymer that consists of repeating units of D-glucuronic acid (GlcUA) and N-acetyl-D-glucosamine (GlcNAc) [6]. In mammals, three isoenzymes are responsible for the synthesis: hyaluronan synthase (HAS) 1, HAS2, and HAS3 [7]. The synthesized HA remains anchored to the cell membrane through the synthase enzymes [8] forming pericellularly [9]. The different synthases have different catalytic activity and produce different molecular-weight HA: HAS3 is more active than HAS2, and HAS1 is less active than HAS2 [10]. The common molecular weight of HA in the human body is above 1000 kDa [8]. It has a role as a lubricant and an ECM component, modulating hydration and osmotic balance in tissues [11]. HA accumulation and alteration in its molecular weight distribution are hallmarks of many pathological conditions, e.g., tumorigenesis [11], human endothelial cell barrier dysfunction [12], and chronic inflammations such as inflammatory lung diseases and rheumatoid arthritis [13]. HAS3 synthesizes medium-molecular-weight (MMW) HA with a molecular mass ranging from 50 to 1000 kDa, while HAS1 and HAS2 produce high-molecular-weight (HMW) HA of more than 2000 kDa [7]. In addition, three other fractions are also classified: low-molecular-weight (LMW) HA is less than 40 kDa, oligomeric HA is less than 10 kDa, and very-high-molecular-weight HA is more than 6000 kDa [14]. In addition to the HA synthesized inherently with different molecular weights, HA can be fragmented by hyaluronidases and reactive oxygen species [5]. HA fragments with different sizes have different biophysical and biochemical properties [15], as well as distinct physiological roles [14,16]. HA is capable of retaining 1000 times more water than its own weight [17], so HA overproduction can lead to edematous swelling of tissues [18]. The hydrodynamic parameters of HA samples vary according to the molecular weight of HA, e.g., the intrinsic viscosity and hydrodynamic radius increase with molecular weight [15,19], which suggests that HMW-HA can hinder the drainage of excess fluid and worsen the edema.

During the pathogenesis of TED, the excess amount of HA contributes to the development of edema and the expansion of orbital tissue volume [20]. Since measuring the HA content of orbital tissues during the clinical course of TED or under the effect of different conditions characteristic of TED is difficult, examining HA production in primary cultures of OFs established from orbital connective tissue is a well-known option as an in vitro model [21]. The details of the TED pathomechanism, including the aspects of HA production during adipogenesis in OFs, are not fully clarified. Studies on OFs usually focus on the quantity and not the quality of HA, although Krieger and Gershengorn [22] have already raised, in 2013, that the size distribution of HA secreted by OFs may be an overlooked aspect of TED pathogenesis. In this study, our aim was to measure the amount and the molecular weight distribution of HA produced by OFs using ELISA and an agarose gel electrophoresis technique, respectively. Primary cultures of OFs established from orbital connective tissues from patients with or without TED were studied under basic and adipogenic conditions.

## 2. Results

### 2.1. The Hyaluronan Production of Orbital Fibroblasts Increases Under Adipogenic Stimuli

**Figure 1:**
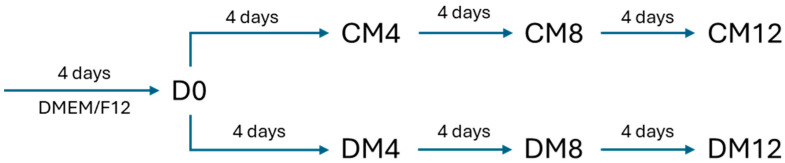
Schematic representation of the experimental setup. DMEM/F12: Dulbecco′s modified Eagle′s medium—high glucose/F12 medium, CM: complete medium composed of DMEM/F12 supplemented with vehicle, DM: differentiation medium composed of DMEM/F12 supplemented with adipogenic cocktail (please see Section 4.2, Tissues and Cell Cultures), D0: initial day after 4 days in DMEM/F12. The numbers after CM and DM indicate the day of sampling.

The experimental setup is shown in Figure 1.

**Figure 2:**
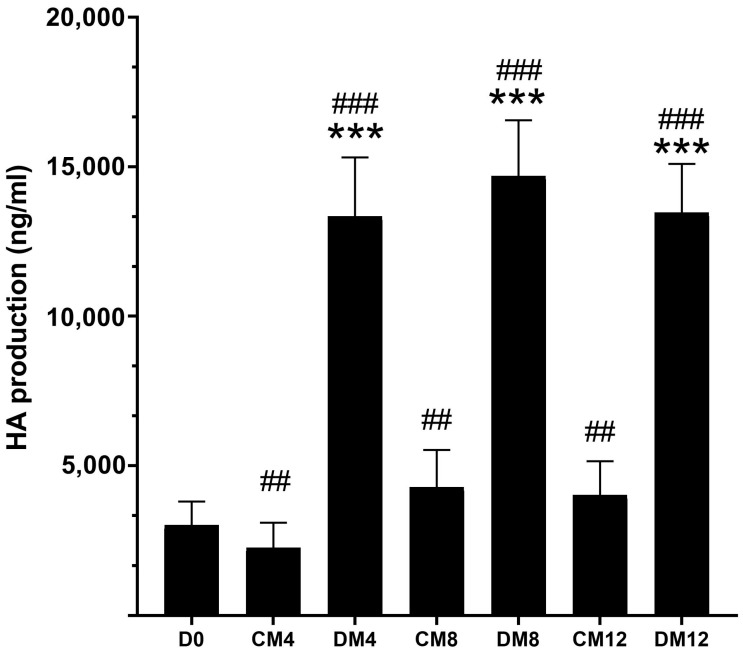
Quantitative determination of HA in the culture media of OFs (n = 6). Data are expressed as mean ± standard error of the mean (SEM). CM: complete medium composed of DMEM/F12 supplemented with vehicle, DM: differentiation medium composed of DMEM/F12 supplemented with adipogenic cocktail, D0: initial day after 4 days in DMEM/F12. The numbers after CM and DM indicate the day of sampling. * means DM compared to CM on respective days, # means DM and CM compared to D0; ^##^ *p* < 0.01, ^###^ *p* < 0.001, *** *p* < 0.001.

The HA content in the culture media of OF cultures established from orbital connective tissues of patients with TED (n = 3) and control patients without TED (non-TED, n = 3) was examined. The basal HA production did not differ in TED and non-TED OFs (*p* = 0.854); the combined results are presented in Figure 2. Compared to the first 4 days from plating, i.e., from day 0 to day 4 during incubation in complete medium (CM), the amount of HA decreased slightly (*p* < 0.01), then increased by days 8 and 12 (*p* < 0.01). In contrast, as a result of adipogenic induction by differentiation medium (DM), a prominent increase was found in the HA production of OFs (*p* < 0.001), which remained at a high level on days 8 and 12 regardless of TED or non-TED origin (*p* = 0.13). In accordance with our previous results [23], substantive adipogenesis was found only in TED-OF cultures. Despite their different adipogenic potential, the extent of the increase in HA production did not differ between TED and non-TED OF cultures under adipogenic stimuli (*p* = 0.314).

### 2.2. The Molecular Weight Distribution of Hyaluronan Produced by TED and Non-TED OFs Shows Different Patterns

**Figure 3:**
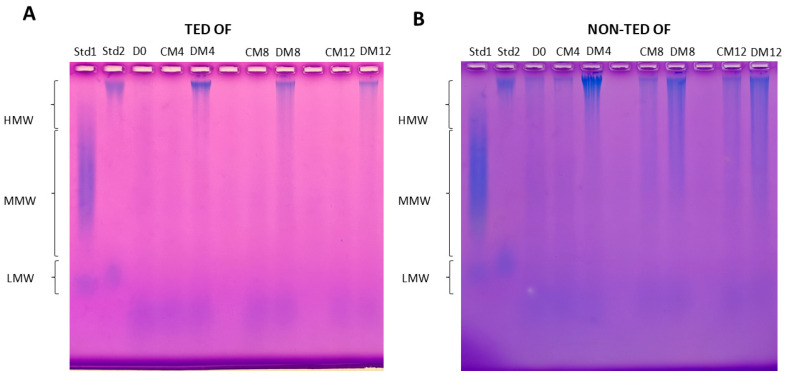
Representative agarose gel electrophoresis results from the culture media of OFs. (**A**) TED OF—lane 1: HMW-HA and ULMW-HA standards, lane 2: MMW-HA and LMW-HA standards, lanes 3–7: samples isolated from the culture media of TED OFs incubated in basic (D0, CM4, CM8, CM12) and adipogenic (DM4, DM8, DM12) conditions, (**B**) non-TED OF—lane 1: MMW-HA and ULMW-HA standards, lane 2: HMW-HA and LMW-HA standards, lanes 3–7: samples isolated from the culture media of non-TED OFs incubated in basic (D0, CM4, CM8, CM12) and adipogenic (DM4, DM8, DM12) conditions. Std: standard, CM: complete medium composed of DMEM/F12 supplemented with vehicle, DM: differentiation medium composed of DMEM/F12 supplemented with adipogenic cocktail, D0: initial day after 4 days in DMEM/F12. The numbers after CM and DM indicate the day of sampling.

Since the performed method to measure HA concentration did not provide information about its molecular weight distribution, HA was further analyzed using agarose gel electrophoresis to show if there were differences in the molecular weight distribution of HA produced by TED OFs and non-TED OFs. Representative results are shown in Figure 3.

**Figure 4:**
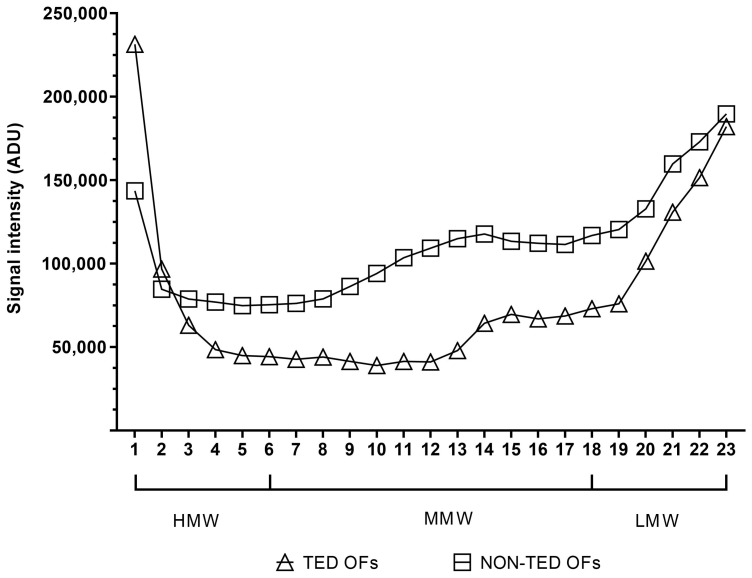
Molecular weight distribution of HA produced by TED (n = 3) and non-TED OF cultures (n = 3) on the initial day (D0), i.e., after 4 days in DMEM/F12. Data are expressed as means. ADU: arbitrary densitometry units.

**Figure 5:**
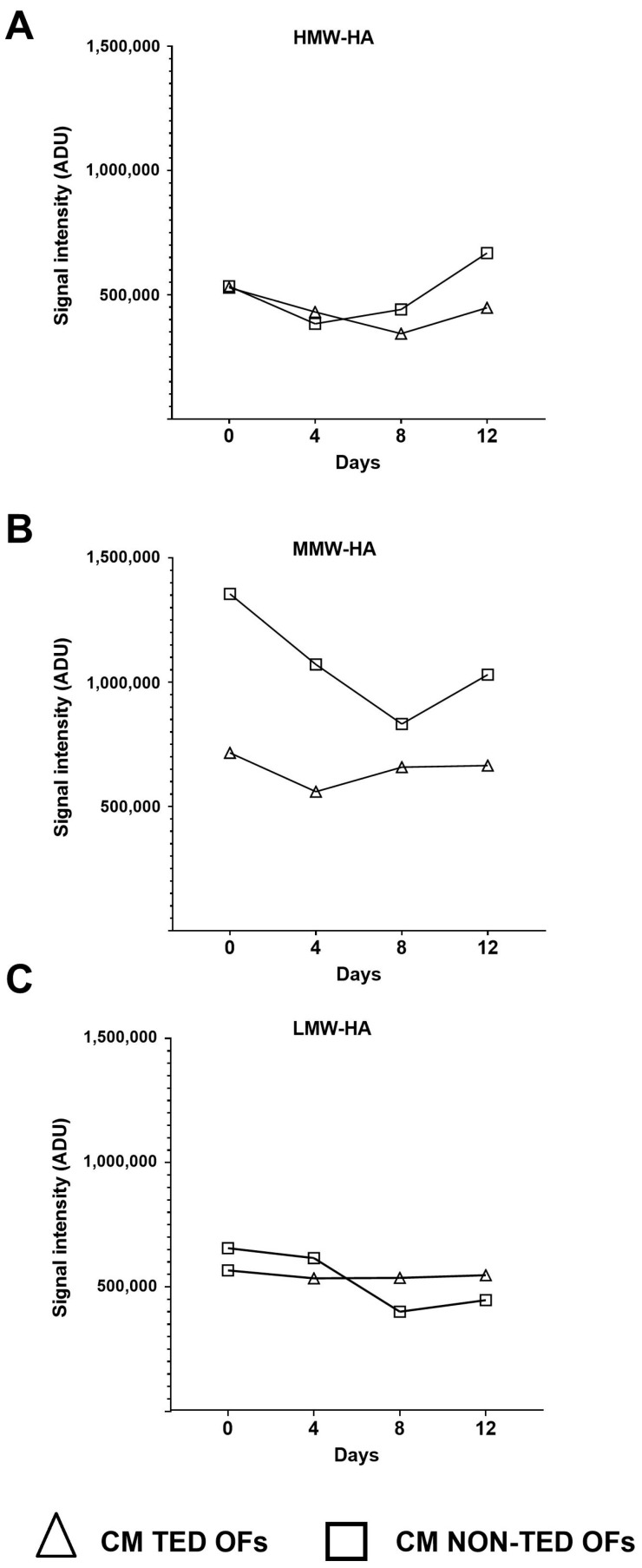
Density values of HMW-HA (**A**), MMW-HA (**B**), and LMW-HA (**C**) over time in the culture media of TED (n = 3) and non-TED OF cultures (n = 3). Data are expressed as means. CM: complete medium composed of DMEM/F12 supplemented with vehicle, ADU: arbitrary densitometry units.

First, the molecular weight distribution of HA produced by TED and non-TED OFs was compared in their basic state (Figure 4). Although the amounts of HA produced by TED and non-TED OFs were not different, the culture media of non-TED OF cultures contained more MMW-HA than did the TED OFs (*p* < 0.001); in the quantity of HMW-HA and LMW-HA, no differences were found (*p* = 0.974 and *p* = 0.437, respectively).

n and the changes over time in the density values, which represent the different molecular weight HA under basic (i.e., unstimulated) conditions, were compared (Figure 5). Origin dependent differences (*p* < 0.001) were found: in TED OFs, all three fractions of HA (HMW-HA, MMW-HA, and LMW-HA) remained unchanged over time, while in the non-TED OFs, HMW-HA decreased on day 4 and increased on day 12 (*p* = 0.006 and *p* = 0.014, respectively), MMW-HA decreased in all studied time points (day 4: *p* < 0.001, day 8: *p* < 0.001, day 12: *p* < 0.001), and LMW-HA decreased on days 8 and 12 (*p* < 0.001 and *p* < 0.001, respectively) compared to the values measured on the initial day (day 0).

### 2.3. The Molecular Weight Distribution of Hyaluronan Produced Under Adipogenic Stimuli

**Figure 6:**
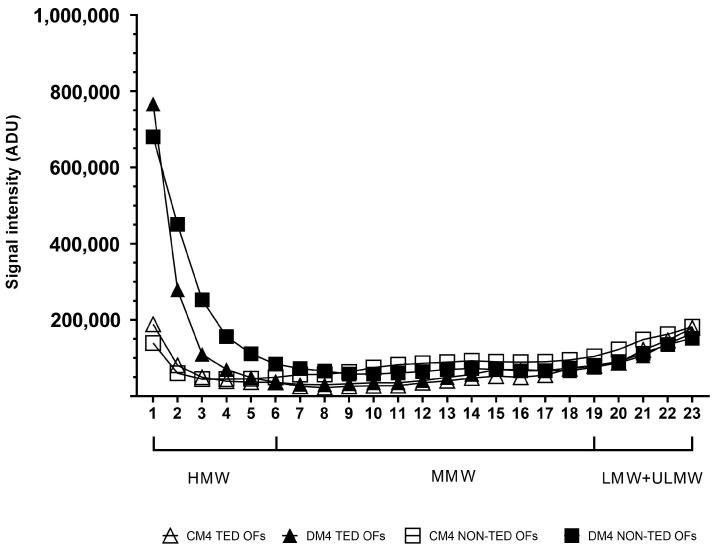
Molecular weight distribution of HA in the culture media of TED (n = 3) and non-TED (n = 3) OF cultures under basic conditions (CM4) and after adipogenic stimuli (DM4) on day 4. Data are expressed as means. CM: complete medium composed of DMEM/F12 supplemented with vehicle, DM: differentiation medium composed of DMEM/F12 supplemented with adipogenic cocktail, ADU: arbitrary densitometry units.

After four days of adipogenic stimuli (Figure 6) there was a marked elevation in HMW-HA in the samples from both TED (*p* < 0.001) and non-TED (*p* < 0.001) OF cultures, while there was no change in the density values of MMW-HA fragments produced by TED (*p* = 0.686) and non-TED OFs (*p* = 0.507). Similar results were observed regarding LMW-HA in TED (*p* = 0.799) and non-TED OFs (*p* = 0.368).

**Figure 7:**
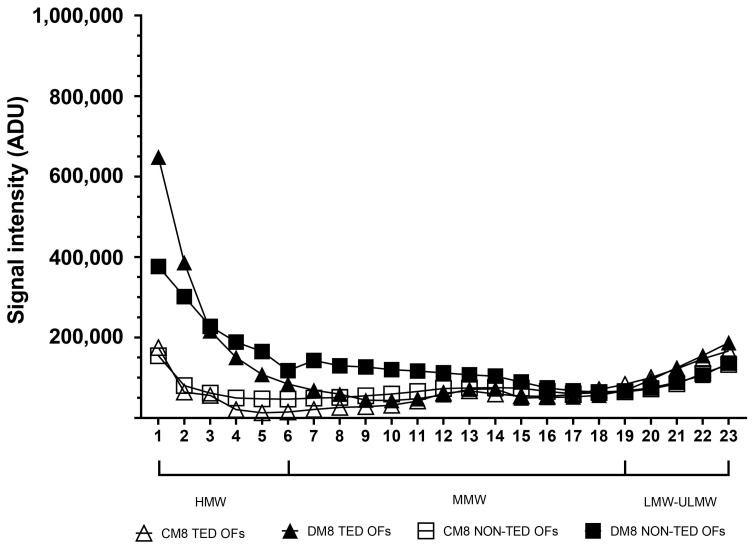
Molecular weight distribution of HA in the culture media of TED (n = 3) and non-TED (n = 3) OF cultures under basic conditions (CM8) and after adipogenic stimuli (DM8) on day 8. Data are expressed as means. CM: complete medium composed of DMEM/F12 supplemented with vehicle, DM: differentiation medium composed of DMEM/F12 supplemented with adipogenic cocktail. ADU: arbitrary densitometry units.

On day 8 of adipogenic stimulation (Figure 7), an increase was seen in HMW-HA in both TED (*p* < 0.001) and non-TED OF cultures (*p* < 0.001). Regarding MMW-HA, only non-TED OFs responded with increased production compared to unstimulated cultures (*p* < 0.01). TED and non-TED OF cultures did not differ in their response in LMW-HA secretion (*p* = 0.934 and *p* = 0.952, respectively).

**Figure 8:**
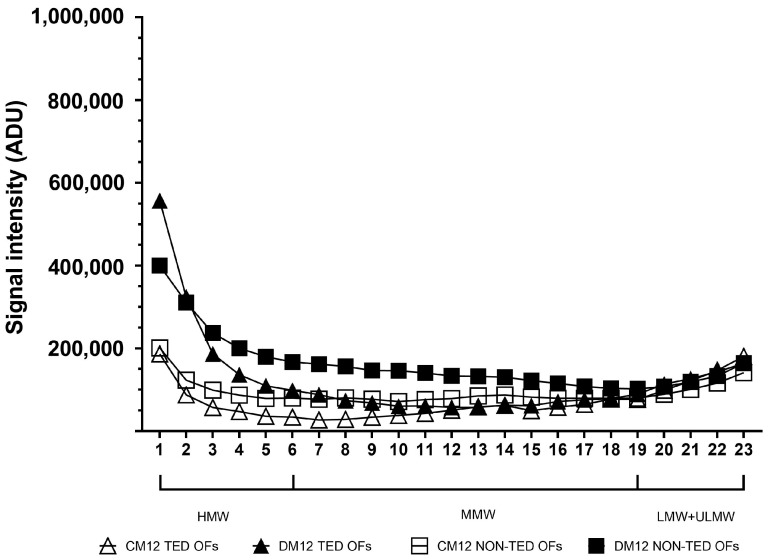
Molecular weight distribution of HA in the culture media of TED (n = 3) and non-TED (n = 3) OF cultures under basic conditions (CM12) and after adipogenic stimuli (DM12) on day 12. Data are expressed as means. CM: complete medium composed of DMEM/F12 supplemented with vehicle, DM: differentiation medium composed of DMEM/F12 supplemented with adipogenic cocktail. ADU: arbitrary densitometry units.

On day 12 of adipogenic stimulation (Figure 8), HA distribution resembled that of day 8.

**Figure 9:**
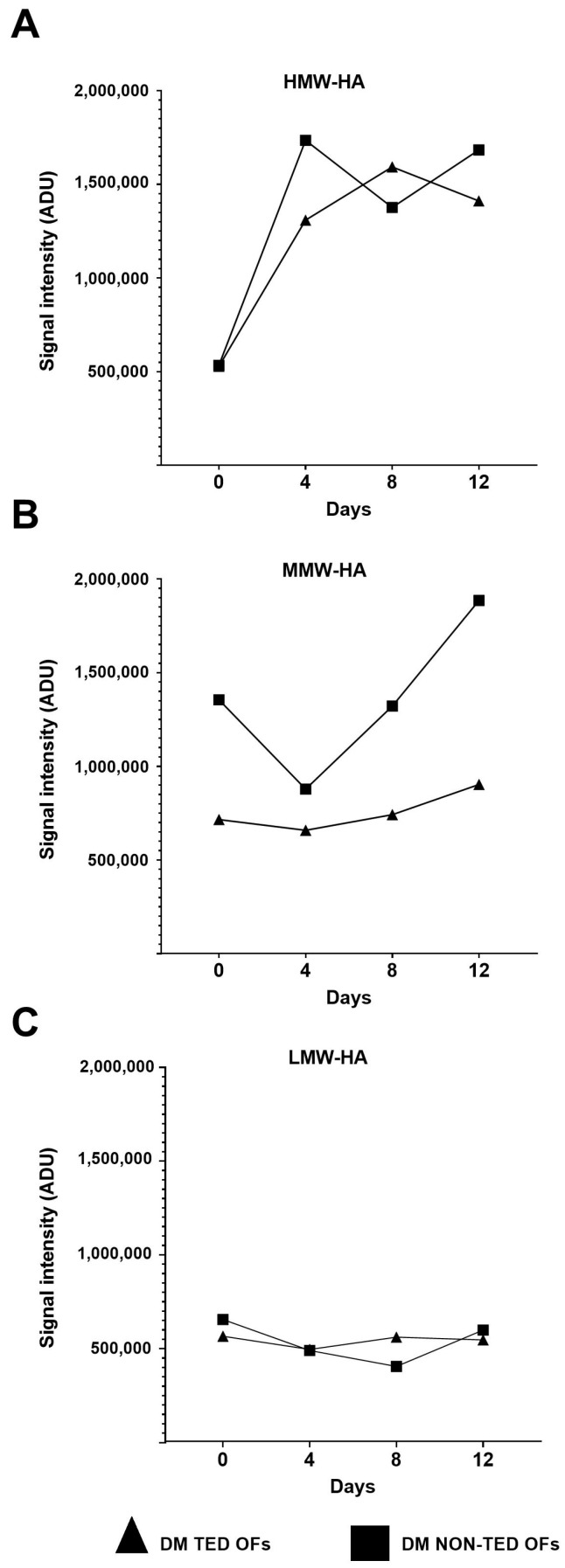
The changes in the density of HMW-HA (**A**), MMW-HA (**B**), and LMW-HA (**C**) over time with and without adipogenic induction at different time points in the culture media of TED (n = 3) and non-TED (n = 3) OF cultures. Data are expressed as means. DM: differentiation medium composed of DMEM/F12 supplemented with adipogenic cocktail. ADU: arbitrary densitometry units.

The time course of production of different molecular weight fractions throughout adipogenesis induction is shown in Figure 9. After a sharp increase between day 0 and day 4 in both the TED and non-TED OFs, from day 4 to day 8, HMW-HA increased (*p* < 0.01) in the TED OFs and decreased in non-TED OFs (*p* < 0.01); then, it did not change significantly until day 12. While MMW-HA did not change in the TED OFs, in the non-TED OF cultures, a sharp increase was seen after day 4 (*p* < 0.01) and again from day 8 to day 12 (*p* < 0.05). No differences were seen in the densities representing LMW-HA at the different time points.

## 3. Discussion

In the pathogenesis of TED, autoantibodies against the TSHR, cytokines, and growth factors produced by infiltrating immune cells lead to the activation of OFs in the orbit [24]. The results of this activation are the accumulation of HA and the differentiation of OFs into adipocytes or myofibroblasts [3]. In our previous study, it was confirmed that myofibroblast differentiation triggered by TGF-β acted toward HA accumulation in the pericellular coat [25]. Others have shown HA accumulation during adipocyte differentiation of OFs [4]. HA is a main component of the ECM in connective tissues and is abundant in the skin, cartilage, brain, and synovial fluid [5]. Due to its properties, HA is widely used for medical, pharmaceutical, and cosmetic applications. The water binding capacity of a HA molecule is related to its structure, conformation, and ionic nature [26]. HA-bound water has a well-known role in tissue edema. In infarcted myocardial tissue, the accumulation of HA contributes to interstitial edema [18]. Other studies reveal that alveolar and interstitial accumulation of HA leads to edema and impaired lung function [27]. In the lymphedematous model in mice, the failures of the lymphatic system are characterized by severe swelling due to HA accumulation [28]. In addition, in rat experimental grafts, the measured relative water content increases and correlates positively with HA accumulation [29]. Kaichi and colleagues examined MRI images, and they found that the water fraction in orbital fat is higher in TED patients compared to healthy controls. Histological examination reveals that edema developed due to elevated glycosaminoglycan content in the orbital fat and extraocular muscles of TED patients [30]. A common MRI approach to determine the quantity of water in tissues is short tau inversion recovery (STIR) images, which enhance the signal from water-containing tissues. Active inflammation is characterized by edema in the orbit, and the water appears as a bright signal on the images [31]. Mayer and colleagues concluded that the clinical activity of TED correlates with the maximal signal intensity of the most inflamed tissue in these images [32]. In a published case of active TED when diuretic treatment resulted in rapid clinical improvement, STIR confirmed that the water content of the orbital connective tissue decreases substantially [33]. These data provide evidence that HA, which accumulates during the course of TED, contributes significantly to the development of tissue edema.

In accordance with others [4], we found elevated HA production in OFs after adipogenic induction, regardless of whether the cells originated from TED or control patients. Since only TED OFs (as opposed to non-TED OFs) can differentiate into adipocytes in culture after adipogenic induction [23,34], we have hypothesized that the adipogenic condition itself can lead to HA buildup in the culture media of OFs, regardless of the differentiation potency of the cells. Thus, we aimed to study whether there were any differences in the molecular weight distribution of HA produced by TED and non-TED OFs under adipogenic stimuli according to their different potential to differentiate. A shift was found towards MMW-HA in the culture media of non-TED OFs, even in the unstimulated state, compared to TED OFs, a difference becoming more pronounced after 8 and 12 days in adipogenic conditions. Although HMW-HA is the most abundant form of HA responsible for the HA accumulation under adipogenic stimuli in both TED and non-TED OF cultures, in non-TED OF, the increasing amount of MMW-HA also contributes to that. Increasing HA concentration in the culture media of OFs may be due to the increased HAS2 expression under adipogenic stimulus [4]. Theoretically, the decreased hyaluronidase expression or activity during adipocyte differentiation may contribute to the predominance of HMW-HA in TED OFs. However, further studies are needed to prove this hypothesis. Comparing the expression pattern of HA-metabolizing enzymes to the molecular size distribution of secreted HA in OFs is one of our future directions. To our knowledge, there are no published data available on the changes in HA metabolism in detail under adipogenic stimuli using human cells. The predominance of HMW-HA in OFs undergoing adipogenesis was previously shown using polyacrylamide gel electrophoresis [22]; however, different conditions were used, and the molecular weight distribution of produced HA was not compared by the origin of the OFs.

The role of HA at the cellular level depends on the size of the HA fragments. Usually, HA is present in high-molecular-weight form above 1000 kDa [35], mostly in the pericellular coat [9]. HMW-HA is a key component of the ECM that plays a major role in the organization of tissue structures, activating signaling pathways and regulating proliferation and migration through interactions with cell-surface receptors and binding molecules [5]. HMW-HA has antiangiogenic and anti-inflammatory properties [35]; upregulation of the synthesis in lung epithelial cells protects against acute lung injury, reduces apoptosis and facilitates survival [36], and is a natural immunologic depressant [19]. In addition, it is the major component of the ECM of the central nervous system and has direct protective capability [37]. In kidney injury and disease, it helps maintain pericellular stability and promotes regeneration [38]. For the reasons mentioned above, HMW-HA appears to have many beneficial and protective effects for cells. Under inflammatory conditions or injury, the lower-molecular-weight forms of HA appear by the breakdown of HMW-HA. MMW-HA has a role in adhesion, proliferation, wound closure, and inflammatory processes [14], and it attracts immune cells to the site of the injury [14,39]. Moreover, in mouse kidney epithelial cells, it directly stimulates the intercellular adhesion molecule 1 (ICAM-1) and vascular cell adhesion molecule 1 (VCAM-1) through NF-κB [40]. LMW-HA has been shown to act as a proinflammatory factor [35] and promote angiogenesis [41].

Based on our current observations, OFs respond with HA overproduction to adipogenic conditions, and the HA produced by TED OFs under adipogenic stimulation is due to the increase in the amount of HMW-HA. This molecular weight distribution may have local unfavorable effects due to the hydrodynamic properties of HMW-HA, which may exacerbate tissue swelling in vivo. In a closed and limited space like the human orbit, the many positive physiological effects of HMW-HA are overridden by the space-occupying matrix expansion. In addition, based on our results observed in non-TED OFs, the higher proportion of MMW-HA can contribute to the inability of non-TED OFs to differentiate into adipocytes [42], but this assumption needs to be confirmed in cells of human origin. We speculate that treatment strategies, either by exogenous hyaluronidase treatment or by altering endogenous hyaluronidase expression that could reduce the proportion of HMW-HA, may have a positive influence on the course of TED, but the potential unfavorable effects of MMW-HA and LMW-HA remain uncertain.

The novelty of the current approach is that it provides insight into the molecular weight distribution of HA produced by OFs under basic and adipogenic conditions. Besides the adipocyte differentiation and lipid accumulation in the TED orbit, increased HA production and a shift towards the predominance of HMW-HA contribute to increased pressure within the orbit via matrix expansion and increased water binding, respectively.

There are several limitations of our study. Of the available methods of HA molecular weight distribution analysis, i.e., size-exclusion chromatography (SEC) [43] or SEC with multiangle light scattering (SEC-MALS) [44] and agarose gel electrophoresis [45], one limitation of the study is that our method is restricted to agarose gel electrophoresis and our results were not validated using a different analytical method. The inherent difficulty of the HA gel electrophoresis remains a limitation; instead of the clear bands seen in protein electrophoresis, the different molecular weight fractions do not fully separate, and a rather blurred image must be evaluated. The technical approach, tediously refined by us, does overcome many difficulties, and though it is not perfect, we believe that it provides better separation than previous techniques. Although most of the differences described are unequivocal, e.g., the differentiation potential of OFs with different origins, increased HA production under adipogenic stimuli, and the higher ratio of MMW-HA in the culture media of non-TED OFs, enrolling more primary cultures in the study would have strengthened the conclusions.

The size of the HA molecules present in the connective tissue matrix is a major determinant of its behavior and may have consequences in disease conditions. Normal healthy tissues contain mostly HMW-HA, and HA fragmentation is usually connected to pathological changes [46]. In the orbital connective tissue, accumulation of HA with any molecular weight can worsen the symptoms, but increasing the viscosity of retained fluid due to a higher amount of HMW-HA can exacerbate the edematous swelling. The agarose gel electrophoresis method optimized for HA from cell culture media may be suitable for the investigation of HA molecular weight distribution in the in vitro models of other pathological conditions or diseases in which the role of increasing HA production or HA fragmentation was described (e.g., pulmonary fibrosis, epithelial cell permeability, tumor development, metastasis formation).

## 4. Materials and Methods

### 4.1. Materials

The DMEM:F12, Medium 199, stable glutamine supplement (SG), Dulbecco’s phosphate-buffered saline without calcium and magnesium (DPBS), fetal bovine serum (FBS), and penicillin/streptomycin (P/S) were purchased from Biosera (Nuaille, France). The TrypLE Express was purchased from Gibco (Thermo Fisher Scientific, Waltham, MA, USA). The biotin, pantothenic acid, dexamethasone (DEX), isobutylmethylxanthine (IBMX), insulin, rosiglitazone (ROSI), triiodothyronine (T3), dimethyl-sulfoxide (DMSO), formaldehyde solution (37%), Proteinase K, ethanol, Tris/Borate/EDTA (TBE) buffer, Oil Red O, and Bromophenol blue were purchased from Sigma Aldrich (St. Louis, MO, USA). The Bioline agarose was purchased from Meridian Bioscience (Cincinnati, OH, USA). The HA with different molecular weight ranges was purchased from Tocris (Bio-Techne, Minneapolis, MN, USA).

### 4.2. Tissues and Cell Cultures

Primary OF cultures were established from orbital connective tissue removed during the decompression surgery of three patients with TED and from orbital connective tissue removed during enucleation surgery of three patients without thyroid disease. Tissues for TED OF cultures (n = 3) were obtained during decompression surgery from patients (two females and one male) with inactive TED (CAS ≤ 3). The mean age was 52 years. The mean TED duration from disease onset was 4 years. All patients had received thyreostatic medication and glucocorticoid therapy in the past. One patient received orbital irradiation; no glucocorticoid treatment or irradiation was performed during the 12 months before orbital surgery. The sources of control OF cultures (non-TED OFs; n = 3; one female and two males) were enucleation surgeries performed in patients with orbital diseases. The mean age of non-TED patients was 57 years. All patients provided written informed consent. Primary OF cultures were established as described in detail in our previous study [1]. The OF cultures were maintained in Medium 199 containing SG and a 1% P/S solution, supplemented with 10% FBS.

For the experiments, 40,000 cells/well, the cells corresponding to post-confluent density were plated into 24-well plates and maintained for 4 days. For adipogenic induction, the cells were maintained in a differentiation medium (DM) containing 1.7 µM insulin, 33 µM biotin, 17 µM pantothenate, 1 nM T3, and 10 µM ROSI in a DMEM:F12 medium supplemented with 250 µM IBMX and 10 µM DEX for the first 4 days only. For control cultures, the DMEM:F12 supplemented with vehicle (complete medium—CM) was used. The experiment lasted 12 days, and samples were collected at the initial time point (day 0) and on days 4, 8, and 12 (Figure 1).

For measuring the fibroblasts’ differentiation potential towards adipocytes, Oil Red O staining of lipids accumulating in differentiating cells was performed as described in our previous study [23].

### 4.3. HA Measurement

The concentration of HA in the culture media was measured using the DuoSet Hyaluronan ELISA kit (R&D Systems, Minneapolis, MN, USA), according to the manufacturer’s instructions using a DTX 880 Multimode Detector (Beckman Coulter, Brea, CA, USA).

### 4.4. HA Isolation from Cell Culture Media

The culture media of the cells were sampled every 4 days and stored at −20 °C until the measurements were carried out. For isolation of the HA, 1 mg/mL Proteinase K solution was pipetted into each sample per mL, then the samples were incubated at 60 °C for 4 h. In the next step, 4 mL of 96% pre-chilled ethanol was added to the medium per mL to precipitate HA from the solution, then incubated at −20 °C overnight. The next morning, the samples were centrifuged at 13,400 RPM for 10 min at room temperature. The supernatant was discarded. The pellet was washed with 75% pre-chilled ethanol and centrifuged again at 13,400 RPM for 10 min at room temperature. The culture media was discarded, and the pellet was air-dried. The isolated sample was suspended in 30 µL of TBE buffer. The samples were then stored at 8 °C overnight.

### 4.5. HA Separation by Agarose Gel Electrophoresis

We further modified an agarose gel electrophoresis method based on Cowman’s technique [25,47]. We prepared 80 mL of a 1.5% agarose gel in TBE buffer for the separation. Before pouring the gel, the agarose solution was cooled to approximately 40 °C, ensuring the uniformity of the gel surface. The gel solidified in about 30 min. To remove impurities, a pre-run was performed at a constant 50 V for 11 h. The TBE buffer was replaced after 5.5 h, and the pre-run was continued immediately. After the pre-run, the gel was left in the buffer overnight. The next morning, to run the samples, fresh TBE buffer was poured so that it covered the gel with a maximum thickness of 3–4 mm, since too much or too little buffer can impair the separation of HA molecules.

The prepared isolated HA samples were mixed with 15 µL of 0.02% bromophenol blue. Since the volume of the prepared samples exceeded the capacity of the gel pockets, we added our samples in several steps. First, we applied a 25 µL sample, then ran it for a few minutes at 20 V in order to load the first portion of the sample into the gel; then we added the remaining amount. A total of 30 min was needed for the entire sample to get into the gel. This was followed by a constant 40 V run for 3.5 h. After the run was completed, the gel was placed in 30% ethanol for 1 h in the dark. The gel was placed in Stains All solution (0.005% dye in 50% ethanol) and stained overnight in the dark. The next day, the gel was placed into 10% ethanol for 1 h to remove excess dye from the gel matrix. Then, the gel was placed in distilled water on the benchtop for 30 min. Detection was performed using an LED transilluminator and photographed.

### 4.6. Evaluation of Molecular Weight Distribution

**Figure 10:**
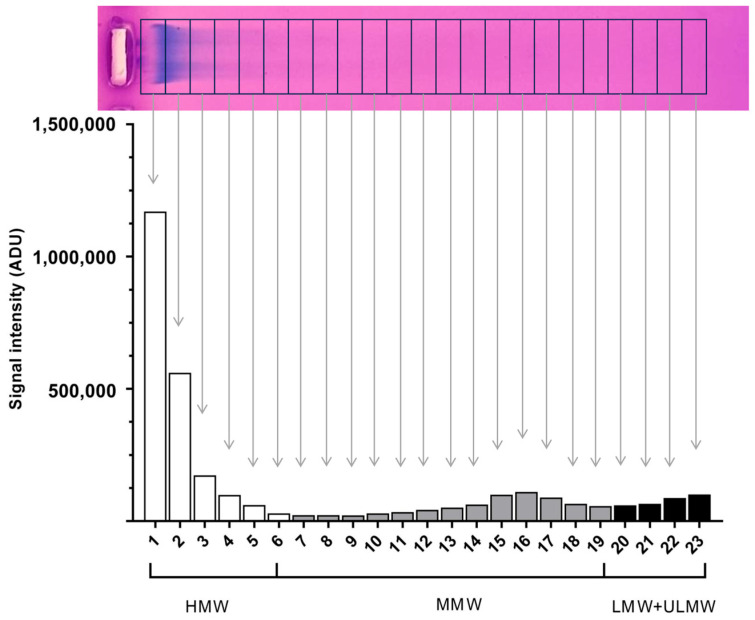
Evaluation HA of molecular weight distribution. The gel photo on the top shows the positioning of the 23 cells for evaluation. Below, the respective densitometric readings are shown. HMW-HA: cells 1 to 6, MMW-HA: cells 7 to 19, LMW-HA and ULMW-HA: cells 20 to 23. ADU: arbitrary densitometry units.

The results of the HA gel electrophoresis were evaluated using Image Studio Digits 5.2 (LI-COR Biotechnology, Bad Homburg, Germany) software. In order to make the resulting density curve evaluable for statistical analyses, we divided the profiles of the run, starting from the highest-molecular-weight (slowest running) to the smallest-molecular-weight (fastest running) sections, into 23 equal cells (Figure 10). The discrete densitometric readings belonging to individual cells were used for comparison. Based on the hyaluronan MW standards (Figure 3, lanes 1 and 2), the size distribution of HA was divided into 3 groups: HMW-HA > 950 kDA (cells 1 to 6), MMW-HA 75-1000 kDA (cells 7 to 19), and LMW-HA < 40 kDA (LMW-HA and ULMW-HA, cells 20 to 23). During the evaluation, the readings of 3 TED and 3 non-TED OF cultures were used. The densitometric data were visualized with GraphPad Prism 8 (Boston, MA, USA).

### 4.7. Statistical Analyses

Statistical analysis of the research data was performed by using STATISTICA version 13.5.0.17 software (TIBCO Software Inc., Palo Alto, CA, USA). A repeated-measures analysis of variance (ANOVA), followed by the Fisher LSD post hoc test, was performed to evaluate the differences, with time or the presence of adipogenic stimuli as the within-subject factor and origin and molecular weight as between-subjects factors. Statistical significance was set at the 5% level (*p* < 0.05).

## Data Availability

The raw data supporting the conclusions of this article will be made available by the authors upon reasonable request.
